# Clinicopathological studies of gastrointestinal tract disorders in sheep with parasitic infection

**DOI:** 10.14202/vetworld.2015.29-32

**Published:** 2015-01-09

**Authors:** Sarvan Kumar, K. K. Jakhar, Satyavir Singh, Sandeep Potliya, Kailash Kumar, Madan Pal

**Affiliations:** 1 Department of Veterinary Pathology, Lala Lajpat Rai University of Veterinary & Animal Sciences, Hisar, Haryana, India; 2 Department of Veterinary Parasitology, Lala Lajpat Rai University of Veterinary & Animal Sciences, Hisar, Haryana, India; 3 Department of Veterinary Surgery and Radiology, Lala Lajpat Rai University of Veterinary & Animal Sciences, Hisar, Haryana, India; 4 Department of Veterinary Gynaecology and Obstetrics, Lala Lajpat Rai University of Veterinary & Animal Sciences, Hisar, Haryana, India

**Keywords:** clinico-pathology, gastrointestinal tract disorders, parasitic infection, sheep

## Abstract

**Aim:**

This study was envisaged to elucidate the parasitological aspects of gastrointestinal tract (GIT) disorders of sheep.

**Materials and Methods:**

Fecal, blood and serum samples collected from 31 sheep/lambs of Sheep Breeding Farm, Lala Lajpat Rai University of Veterinary & Animal Sciences, Hisar.

**Results:**

Of 25 cases, strongyle eggs (12 cases, 48%) were a major infection, followed by *Strongyloides* spp. (8 cases, 32%) and *Moniezia* spp. (5 case, 20%). In one case, massive infection of strongyle particularly *Haemonchus contortus* and *Moniezia* spp. was observed. All these animals were found negative for hemoprotozoan parasites in blood smear examination. Hematological studies revealed that significantly decreased values of hemoglobin (Hb), packed cell volume (PCV) and total erythrocytic count (TEC). Absolute leukocytic count revealed significant leukocytosis due to neutrophilia, lymphocytosis, monocytosis and eosinophilia. Serum biochemical profiles of diarrheic sheep/lambs in present study were significant decrease in values of total protein, serum globulin, glucose where as significant increase in the albumin: Globulin ratio, aspartate aminotransaminase (AST), alanine aminotransaminase (ALT), alkaline phosphatise (ALKP) and bilirubin.

**Conclusions:**

From the present study, it is reasonable to conclude that major parasitic infection of sheep/lamb observed was strongyle, followed by *Strongyloides* spp. and *Moniezia* spp. Hemato-biochemical studies revealed significant leukocytosis and increase in AST, ALT, ALKP and bilirubin.

## Introduction

Agriculture is the mainstay of Indian economy, where in agriculture and allied sector like, livestock, dairying and fisheries contribute about 15.18% of Gross Domestic Product [1]. Contribution of livestock sector to the food basket in the form of milk, egg and meat is of significance in fulfilling of animal protein requirement of ever and fast growing human population. In rural India where over 15-20% families are landless and about 80% of the land holders belong to the category of small and marginal farmers, livestock is the main source of livelihood. In the absence of fertile lands and assured irrigation which are controlled by a small population of rich farmers and lack of employment in the industrial and service sectors, most of the rural families belonging to socio-economically weaker sections of the society maintain different species of livestock to supplement their income. Although the landowners prefer cattle and buffaloes, the landless prefer to own sheep, goat and poultry. With the policy of the State Animal Husbandry Department to extend free breeding, vaccination and veterinary services and permit free grazing on community lands, the farmers were encouraged to expand their herd size without any major financial burden [2]. This has probably been the reason for the presence of the world’s largest livestock population in India. India ranks second in sheep population (71.56 million) in the world [3]. Of this 0.63 million sheep account from Haryana [4]. Although safe and effective treatment and control methods exist for the most internal and external parasites, many animals continue to suffer from preventable parasitic infections. Geographical location, housing conditions and species play a role in which parasites are likely to be a problem. Internal and external parasites can cause great discomfort, transmit disease to animals and significantly interfere with the growth and performance of animals.

Hematology and serum biochemistry of infected animals is very sensitive indicators for the degree of hepatic damage and the parasitic infection severity, in which liver damage upsets the vital metabolic processes for normal health and optimum productivity of the animal.

The purpose of this study was to overview the correlation between clinicopathology in the gastrointestinal tract (GIT) disorders due to some parasitic infection and its effect on hematology and clinical biochemistry in sheep.

## Materials and Methods

### Ethical approval

The study was conducted after the approval of the Institutional Animal Ethics Committee.

### Collection of sample

Proposed study was conducted on 31 sheep/lambs including 6 healthy and 25 diseased sheep/lambs affected with GIT disorders. Samples of feces, blood and serum for research was collected from Sheep Breeding Farm, Lala Lajpat Rai University of Veterinary & Animal Sciences, Hisar.

### Faecal examination for parasitic eggs and ova

Fecal samples were collected directly from the rectum from clinical cases of sheep/lamb having GIT disorders. These fecal samples were examined for the presence of helminthic ova, both heavy and light, and coccidial oocysts. For examination, floatation and sedimentation methods were employed [5].

### Hematological examination

Hematological studies *viz*., Hb, TLC, differential leukocytic count, TEC, PCV, erythrocytic sedimentation rate (ESR), mean corpuscular volume (MCV), mean corpuscular Hb and mean corpuscular Hb concentration (MCHC) were estimated following the method of Benjamin [6] within the 6 h of blood collection.

### Serum biochemical examination

Serum biochemical parameters were determined by using Technicon Ames RA-50 chemistry analyzer using diagnostic kits of Bayer [7]. The levels of the following plasma constituents were determined: Total protein (TP), albumin, total bilirubin and enzyme activities of alkaline phosphatase (ALKP), alanine aminotransferase (ALT) and aspartate aminotransferase (AST). The gamma-globulin levels were also measured and the albumin: Globulin ratio was calculated.

### Statistical analysis

The Student’s t-test using SPSS statistics 17.0 software (IBM Corporation, New York, USA) was applied to statistically analyze the results obtained with different study groups.

## Results

The results of parasitic findings in 31 cases (6 healthy animals and 25 animals showing GIT disorders) of sheep/lambs are; maximum infection was in sheep of age group 2-5 months and least in age group of >1 year. Out of 25 cases, strongyle eggs (12 cases, 48%) was a major infection, followed by *Strongyloides* spp. (8 cases, 32%) and *Moniezia* spp. (5 case, 20%), respectively. In one case, mixed massive infection of strongyle particularly *Haemonchus contortus, strongyloides* spp. and *Moniezia* spp. were observed. All these animals were found negative for hemoprotozoan parasites in blood smear examination.

**Figure-1:**
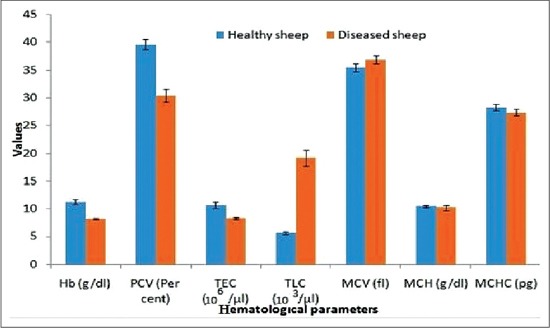
Histograms representing hematological parameters of healthy and diseased sheep.

Hematological studies of blood collected from diarrheic sheep/lambs revealed significantly (p≤0.05) decreased values of Hb (8.184±0.194), PCV (30.40±1.146) and TEC (8.22±0.249). Absolute leukocytic count revealed significant (p≤0.05) leukocytosis (19.156±1.443) due to neutrophilia, lymphocytosis, monocytosis and eosinophilia. No effect was seen on ESR (0.14±0.004). An analysis of erythrocytic indices revealed no significant change in MCV (36.89±0.719), mean corpuscular Hb (10.22±0.463) and MCHC (27.37±0.587) as shown in Figure-1.

**Figure-2:**
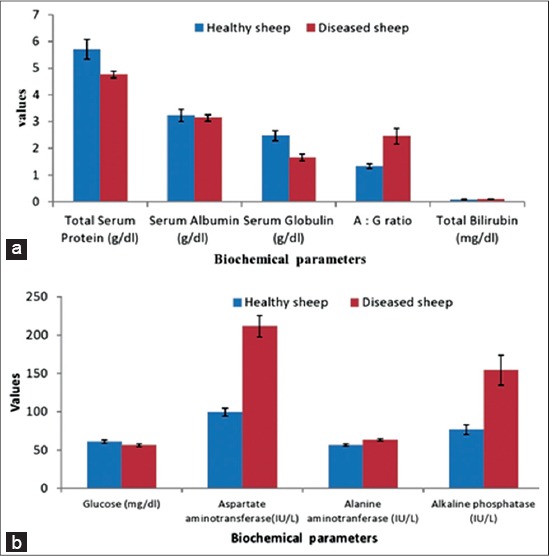
(a,b)Histograms representing serum biochemical parameters of healthy and diseased sheep.

Serum biochemical profiles of diarrheic sheep/lambs in present study showed significant (p≤0.05) decrease in values of TP (4.76±0.118), serum globulin (1.66±0.12030) and serum glucose (56.26±1.725) whereas significant (p≤0.05) increase in the albumin: globulin ratio (2.46±0.29345). The activity of AST (211.6±13.92), ALT (63.34±1.54) and ALKP (154.28±19.662) and total bilirubin (0.107±0.0077) were significantly (p≤0.05) increased in diarrheic sheep/lambs shown in Figures-2a and b. There was no significant difference in hemato-biochemical studies among age group.

## Discussion

Examination of intestinal contents/feces for parasitic isolation revealed maximum infection was in sheep of age group 2-5 months and least in age group of >1 year [8,9]. Mixed parasitic findings revealed maximum cases of mixed infection of strongyle and *Strongyloides* spp. and only one case of mixed infection of strongyle, *Strongyloides* spp. and *Moniezia* spp. These findings were in general, agreement with those reported by Mederos *et al*. and Lashari and Tasawar [10,11]. Prevailing agro-climatic conditions like overstocking of the animals, grazing of young and adult animals together with poorly drained land provide an ideal condition for the transmission of the endoparasites to build up clinical infection of the host. The overall higher incidence of nematodes infection in the areas surveyed could be attributed to lower immunity of hosts as a result of malnutrition. All the livestock in the area under investigation largely depended on grazing in deteriorated range-lands [12].

Hematological studies of blood collected from diarrheic sheep/lambs revealed significantly decreased values of Hb, PCV and TEC. Amongst GI helminths of sheep. *H. contortus* is the predominant species. Main pathological lesion caused by *H. contortus* infection is anemia. Both adult and fourth stage larvae suck blood and in addition, migration of adult and larvae cause hemorrhages into the abomasum. The average blood loss due to *H. contortus* infection is 0.03 ml/parasite/day [13]. The hematological studies in present investigations were in unison with the observation of Dhanlakshmi *et al*. [14], Zaki *et al*. [15] and Purohit *et al*. [16].

Absolute leukocytic count revealed significant leukocytosis due to neutrophilia, lymphocytosis, monocytosis and eosinophilia were in agreement with the observation of Misra *et al*. [17]. The decrease in value of Hb, PCV and TEC might be due to the presence of strongyle infection, which had been recognized as active blood sucker in stomach and intestine and also been observed in present studies.

Serum biochemical profiles of diarrheic sheep/lambs in the present study were significant decrease in values of TP, serum globulin and serum glucose whereas significant increase in the albumin: globulin ratio. These results are in consonance with finding of Maiti *et al*. [18], Pandit *et al*. [19], Dhanlakshmi *et al*. [14] Purohit *et al*. [16], Bordoloi *et al*. [13] and Qamar and Maqbool [20]. Inappetance with the resultant reduction in dietary protein, malabsorption and plasma losses from damaged intestinal mucosa might be the main cause for marked hypoproteinemia. The inflammation of the intestine by development stages of parasites might also due to poor absorption of protein metabolites resulting low level of TP and massive ascites since ascetic fluid contains a large amount of protein [13,14]. Low serum glucose in the diarrheic sheep/lambs might be due to decreased appetite of animals, decreased absorption into the blood stream and rapid absorption and utilization of soluble carbohydrate and lipids from the gut by parasites [21].

The activity of aspartate aminotransferase, alanine aminotransferase and alkaline phosphatase and increased total bilirubin were significantly increased in diarrheic sheep/lambs. Specific hepatic function are greatly affected by a wide variety of the pathological condition of extrahepatic origin specially GIT origin. Similar finding were also reported by Prasanthi *et al*. [22], Zaki *et al*. [15] and Bordoloi *et al*. [13]. Since these enzymes have their function and greatest concentration within the cell increase in enzymatic activities reflect cellular abnormalities which directly related to damage that has occurred to hepatocytes, pathological lesions of intestine and cardiac infarction [16] which were also be evidenced in presented studies. As alkaline phosphatase is widely distributed in the body and found in high concentration in bone (in oestoblast), intestinal mucosa, renal tubules cell, liver and placenta; increase in the alkaline phosphatase value in current studies suggestive to damage to mucosal cells of intestine due to parasitic pathogenesis in GIT disorders.

## Conclusion

Parasitological findings revealed strongyle followed by *Strongyloides* spp. and *Moniezia* spp. Hematological studies evidenced that significantly decreased values of Hb, PCV and TEC. Absolute leukocytic count revealed significant leukocytosis due to neutrophilia, lymphocytosis, monocytosis and eosinophilia. There was no significant change in ESR. Serum biochemical profiles of diarrheic sheep/lambs in present study were significant decrease in values of TP, serum globulin, glucose whereas significant increase in the albumin: Globulin ratio, AST, ALT, ALKP and bilirubin.

## Authors’ Contributions

SK, KKJ and SSG have designed the study and planned the research experiments. SK performed the research experiments. SP, KK and MP help in conducting experiment. All authors read and approved the final manuscript.
